# Intelligence and Verbal Short-Term Memory/Working Memory: Their Interrelationships from Childhood to Young Adulthood and Their Impact on Academic Achievement

**DOI:** 10.3390/jintelligence5020026

**Published:** 2017-06-16

**Authors:** Wolfgang Schneider, Frank Niklas

**Affiliations:** Department of Psychology, University of Würzburg, Würzburg 97070, Germany; niklas@psychologie.uni-wuerzburg.de

**Keywords:** intelligence, short-term memory, working memory, academic achievement, domain knowledge, LOGIC study

## Abstract

Although recent developmental studies exploring the predictive power of intelligence and working memory (WM) for educational achievement in children have provided evidence for the importance of both variables, findings concerning the relative impact of IQ and WM on achievement have been inconsistent. Whereas IQ has been identified as the major predictor variable in a few studies, results from several other developmental investigations suggest that WM may be the stronger predictor of academic achievement. In the present study, data from the Munich Longitudinal Study on the Genesis of Individual Competencies (LOGIC) were used to explore this issue further. The secondary data analysis included data from about 200 participants whose IQ and WM was first assessed at the age of six and repeatedly measured until the ages of 18 and 23. Measures of reading, spelling, and math were also repeatedly assessed for this age range. Both regression analyses based on observed variables and latent variable structural equation modeling (SEM) were carried out to explore whether the predictive power of IQ and WM would differ as a function of time point of measurement (i.e., early vs. late assessment). As a main result of various regression analyses, IQ and WM turned out to be reliable predictors of academic achievement, both in early and later developmental stages, when previous domain knowledge was not included as additional predictor. The latter variable accounted for most of the variance in more comprehensive regression models, reducing the impact of both IQ and WM considerably. Findings from SEM analyses basically confirmed this outcome, indicating IQ impacts on educational achievement in the early phase, and illustrating the strong additional impact of previous domain knowledge on achievement at later stages of development.

## 1. Introduction

The impact of individual differences in psychometric intelligence (IQ) on academic achievement in school and later life has been demonstrated in numerous studies (e.g., [1,2,3]). For instance, Deary et al. [1] carried out a comprehensive prospective longitudinal study of more than 70,000 English children, testing their psychometric intelligence at age 11 and their educational achievement in national examinations in 25 academic subjects at age 16. As a main result, the correlation between a latent intelligence trait and a latent trait of educational achievement was 0.81, indicating that general mental ability assessed at the beginning of secondary education contributed substantially to academic achievement overall. 

Although the impact of IQ on academic achievement has not be disputed in recent research, an important role of short-term memory and working memory capacity for school learning has been highlighted in several studies (see [4,5]). There is evidence that memory components affect academic achievement in addition to psychometric intelligence. In particular, working memory, that is, the ability to process and remember information, is linked to performance in a wide range of cognitive tasks such as reasoning or verbal comprehension [6]. Accordingly, several studies addressed the link between working memory and intelligence, exploring the relationships between short-term memory (STM), working memory (WM), and IQ. 

*Relationships among STM, WM and IQ in Adults*. One problem with this approach is that various models of WM have been suggested in the literature (see reviews by [4,7]). According to the most widely used model originally proposed by Baddeley and Hitch [8], WM (or the central executive) is a domain-general component responsible for the control of attention and information processing. In addition, the storage of information is mediated by two domain-specific STM slave systems, which are responsible for the temporary storage of verbal and visuospatial information. An alternative approach was developed by Friedman and Miyake [9], whose model did not include the distinction between STM and WM and was based only on the assumption that WM is supported by two separate sets of domain-specific resources for handling verbal and visuospatial information. Other authors have argued that STM and WM are hardly distinguishable, and suggested a unitary model of WM [10,11]. For instance, Cowan’s model assumes that WM consists of activated long-term memory representations (or “short-term store”) and a central executive responsible for cognitive control. Within the short-term store, the focus of attention is limited to approximately four items, which means that we cannot activate more than four pieces of information at the same time. 

As noted by Conway, Kane, and Engle [12], it is important to clarify the distinction between the construct WM, as described in various theoretical models, and working memory capacity (WMC). The latter refers to the maximum amount of information an individual can maintain in a particular task that is designed to measure specific aspects of WM. In most studies addressing the relationship between working memory and fluid intelligence, WMC was associated with measures of psychometric intelligence (g-fluid). Recent meta-analyses conducted by different groups of researchers assessing the relationship between WMC and g-fluid in healthy young adults showed a manifest correlation of r = 0.48 [13], although the correlation between latent variables was typically higher: the estimated correlation varied somewhere between r = 0.72 [14] and r = 0.85 [15]. Accordingly, WMC accounted for about half the variance in g-fluid, which is impressive. These findings indicate that WM and intelligence are separable but represent closely related constructs. The incomplete overlap suggests that the two constructs are not isomorphic [12].

*Relationships among STM, WM and IQ in Children.* In comparison, research comparing WM and intelligence in children has produced less robust evidence [16]. Whereas some studies indicated that STM and WM predicted intelligence to about the same extent [17], other studies suggested a comparably stronger impact of WM on intelligence [18]. In a study with young children, Engel De Abreu et al. [19] found that WM, STM, and fluid intelligence were significantly related but separate constructs, and that WM was the best predictor of intelligence. Giofre et al. [16] explored the nature of the relationship between STM, WM, and intelligence in a sample of fourth- and fifth graders, mainly because this age range represents important transitions associated with mind reorganisations (see [20]) and thus seems well-suited to examine the relationship between different aspects of WM and intelligence. Given that several WM, STM, and IQ measures were used in this study, the authors were able to investigate the structure of WM and the relationship between WM and intelligence in detail. As a main result, the measurement model representing WM was in accord with Baddeley’s [21] multicomponent model, distinguishing between a storage STM component and a processing WM component. Moreover, the verbal WM component was more strongly associated with IQ than the verbal STM component, indicating that WM predicts a substantial portion of the variance in IQ when the effect of STM is taken into account. Whereas previous research with children had provided unclear evidence on the strength of the relationship between WM and intelligence, the more recent studies by Giofre et al. [16] and Demetriou et al. [20] indicated that WM and IQ share a substantial portion of the variance, in agreement with findings obtained for adults (e.g., [18]). 

*Relationships between IQ and WM in Predicting Academic Achievement*. Not surprisingly, WM (like IQ) has been shown to be related to learning in school. Individual differences in WMC have important consequences for the acquisition of knowledge and new skills (see [4], for a review). Individual differences in WM tasks not only predicted reading achievement but also math outcomes. Findings from several studies carried out with children showing developmental disorders and problems in the domains of reading and mathematics suggested that low WMC scores contributed significantly to the overall poor performance (e.g., [22,23]). Interestingly, some studies also indicated that the association of low WM scores and learning disability is relatively independent of the level of IQ [22,24].

Given the strong links between WM and learning, several developmental studies explored whether WM is simply a proxy for IQ with regard to academic achievement, or whether WM shares unique links with learning after statistically controlling for IQ. Results from cross-sectional studies provided evidence for both positions: whereas some researchers suggested that IQ is the key factor underlying the relationship between WM and learning (e.g., [25]), others provided evidence for a unique impact of WM on educational achievement in addition to IQ (e.g., [22]). 

In a more recent study, Alloway et al. [26] investigated the respective contributions of WM and IQ to academic achievement within a longitudinal framework. Children’s verbal STM and WM as well as their IQ were first tested in kindergarten when children were about five years of age. They were re-tested six years later on the same memory tasks, a different IQ test, and on standardized measures of learning ability (read, spelling, and math). Using a set of hierarchical regression analyses and literacy as well as mathematics as separate outcome measures, Alloway et al. were able to show that WM had a generally stronger impact on educational achievement than IQ. This finding indicates that WM is not a proxy for IQ but rather represents a dissociable cognitive skill with unique links to learning outcomes. According to the authors, the large contribution of WM to subsequent learning confirms and extends findings from cross-sectional research, suggesting that WM taps more than general intellectual ability. The fact that WM is a more powerful predictor of subsequent academic success than IQ from early formal education on contrasts with the popular view that the link between IQ and learning is greatest (and greater than that of WM and learning) when the child is acquiring new information rather than at later stages when learning gains seem due to the impact of extended practice (see [27]). 

Although the available empirical evidence supports the assumption that WM has a direct influence on academic learning, separately from IQ, methodological issues seem to make a difference in this respect. Lee, Lee Pe, Ang, and Stankov [28] investigated the impact of IQ and WM on algebraic proficiency based on the data of three similar studies with fifth graders. In a first set of analyses, they used hierarchical regression modelling to predict children’s algebraic proficiency, entering IQ in a first step, followed by WM. The same pattern of results was found for all three studies. The data showed that, after controlling for individual differences in IQ, WM explained an additional 5% to 7% of variance in the algebraic proficiency measures, thus basically confirming the assumption that WM is not a proxy for IQ. However, when latent variable structural equation modeling was used based on the same data sets, the outcome was different: whereas the direct path from WM to IQ was significant and substantial, only IQ directly predicted algebraic proficiency. The direct path from WM to algebraic proficiency was not different from zero. 

How should these discrepant findings be dealt with? According to Lee et al. [28], one of the advantages of latent factor analyses is that explicit estimates of measurement error are built into the models. Thus structural relationships among latent constructs are deemed to be free of measurement error and reflect only relationships among the theoretical constructs. In contrast, regression analyses based on observed variables produce estimates of relationships that are confounded by measurement error. Lee et al. [28] concluded from their findings that they are consistent with those of earlier investigations in that working memory substantially contributes to variation in intelligence, which in turn contributes to variation in academic achievement. However, their findings differed from previous outcomes in that no direct effect of WM on academic performance was found. 

*Goals of the Present Study.* Given that the studies on the relationships among WM, STM, and IQ in predicting academic achievement are predominantly cross-sectional in nature and did not come up with consistent results, we decided to reanalyze data from the Munich Longitudinal Study on the Genesis of Individual Competencies (LOGIC) (see [29,30]) to explore the issue further. The LOGIC study started with a sample of around 200 four-year-old children in 1984 and was completed in 2003, when participants were about 23 years of age. The LOGIC sample remained remarkably stable across the 20-year period of data collection, with 152 participants still available at the last measurement point. Assessments covered a broad range of domains of cognitive, social, and personality development (see [29,30]). In the present context, it seemed relevant that measures of short-term memory, verbal as well as fluid intelligence were assessed repeatedly across the course of the study, covering the kindergarten period, middle and late childhood, and also adolescence and young adulthood. Measures of reading, mathematics, and spelling were obtained from second grade on. Whereas reading comprehension was only assessed at the beginning of school and in early adulthood, indicators of math and spelling performance were frequently assessed across the course of the study. 

The major goal of the present reanalysis of the LOGIC data was to explore the predictive power of the STM and IQ variables regarding educational achievement at different occasions. Thus, the new aspect of the present investigation is that predictions were carried out for the same sample at different developmental stages, when participants were 6, 8, 10, 17, and 23 years of age. This enabled us to test the assumption that the relative impact of the IQ variables may be greater at the beginning of school, as compared to later stages of development (see [27]), and that the relationship between IQ and STM is similarly strong in childhood, adolescence, and young adulthood. The inclusion of both verbal and fluid intelligence variables allowed for a test of the assumption that the verbal STM variables are more closely related to verbal IQ, as compared to fluid intelligence. Comparing the outcomes of hierarchical regression analyses with those of latent variable structural equation modeling should provide further evidence on the impact of methodology on findings [28]. We were also able to test the assumption that IQ and STM may have a different predictive power for reading and mathematics in childhood and subsequent developmental stages [31]. A further goal of the present longitudinal approach was that the additional impact of STM and IQ on different aspects of educational achievement could be assessed after preceding educational achievement had been taken into account. Finally, we explored whether a cascade approach—largely used in the developmental literature (cf. [20,16])—in which intelligence mediates the relationship between WM and achievement would fit the data better than our original SEM models in which correlations between the latent WM and IQ variables were specified.

## 2. Method

*Sample.* Originally, 205 4-year-old children from Southern Bavaria were recruited for the study in 1984, and another 25 children joined a year later (see [29]). Children were assessed annually until they reached Grade 6 in 1992. Two follow-up assessments were carried out in 1998 when participants were about 18 years old, and again in 2003 when they were 23 years of age. Our analyses were conducted with a subsample of 215 children (52% male) who had valid assessments for at least one of the assessments and we used the assessments at age 6, 8, 10, 18, and 23 (t1 to t5). A total of 193 children were still available at t3, and 151 participants at t5, indicating that the drop-out rate was rather low for this long period of time (see Table 1).

Participants’ SES score was ascertained on the basis of the highest prestige of the parental occupations (cf. [32]). Such prestige scores are also used in the large educational studies such as PISA, and they are closely associated with education and income of parents.

The scale ranged from 20 (unskilled labor) up to 186.8 (physician). Occupations of 201 households were obtained. The highest prestige score in a household was on average M = 81.2 (SD = 32.1) in this sample.

### 2.1. Measures

#### 2.1.1. Intelligence 

Several psychometric intelligence tests were used during the course of the LOGIC study and included measures of both verbal and nonverbal intelligence. As to the assessment of verbal IQ, the Hannover–Wechsler Intelligence Scale for Preschool Children (HAWIVA) [33] was administered at the beginning of the study. This test is roughly comparable to the WPPSI used in most English-speaking countries. Because of time constraints, only the verbal part of the test was administered. This part consists of three subtests: General Knowledge, Vocabulary, and General Comprehension. 

From the beginning of formal schooling on, the Hamburg–Wechsler Intelligence Scale for School Children (HAWIK) [34] replaced the HAWIVA. The HAWIK test (equivalent to the WISC) can be used for children between 6 and 16 years of age. Only performance on the verbal part of the test is considered in the present analyses. This part consisted of the following subtests: General Knowledge, Vocabulary, General Comprehension, Commonalities, and Numerical Thinking. The HAWIK was administered at age 8 and 10 years. In the two subsequent waves carried out when participants were 18 and 23 years of age, the vocabulary subtest of the Hamburg–Wechsler Intelligence Scale for Adults (HAWIE) was used to assess a core aspect of verbal intelligence. Because of time constraints, it was impossible to include more HAWIE subtests in the LOGIC study. 

To assess nonverbal intelligence, the Columbia Mental Maturity Scale (CMMS) [35] was chosen during the early stages of the study. This test is designed to tap general reasoning abilities of children aged 3 years 6 months to 9 years. Test items consisted of picking out the odd item in a group (i.e., selecting the picture that is different from or unrelated to all the others in a series of three to five drawings). The number of correct answers gives the sum raw score. The results obtained at t1 and t2 were used for the present analyses.

Given that the CMMS measure was no longer appropriate after children entered Grade 4, we replaced it with the German version of the Culture Fair Intelligence Test (CFT), first developed by Cattell and Horn [36] as a measure of nonverbal intelligence from Grade 4 on. The test assesses fluid intelligence and consists of two parallel forms (A and B), each containing four different subtests: The first subtest (Series) requires the identification and completion of series of geometric figures; the second subtest (Classification) requires the classification and differentiation of geometric figures; the third (Matrices) requires children to complete matrix figures; and the fourth (Topologies) requires the identification of proportions and relations of geometric areas. The speed version of the test was used at t3, t4, and t5, when participants were 10, 12, 18, and 23 years old (for more details, see [37]). Reported internal consistency was high for all measures, ranging between 0.80 and 0.93.

Total scores of non-verbal and verbal intelligence were calculated (see also [3]).

#### 2.1.2. Working Memory

Two short-term memory tasks were used to assess working memory capacity (WMC). The first task tapped children’s memory span for words, using a paradigm developed by Case, Kurland, and Goldberg [38]. In this task, 10 different word sets with three to seven items were used. Children were instructed to first listen to the entire tape-recorded set and to reproduce it immediately thereafter. Beginning with the smallest set of three words, children were given two trials for each set size. They were instructed to listen to the entire set and then to repeat the words they heard. Whenever an item set could be correctly reproduced, the next set including n + 1 items was presented. This procedure continued until the children could no longer recall all of the items. Two different dependent measures were calculated. The first word span measure was identical to that used by Case et al. [38]. Participants were given credit for correctly recalling a set of items as long as they could remember all of the words, regardless of order (i.e., unconstrained word span). According to Case and colleagues, this measure ensures that developmental differences in span are not confounded with differences in encoding and preserving information about order. A second measure was the serial word span (words had to be recalled in correct order), which enabled us to explore differences in these two span measures as a function of age. The word span measure was assessed at ages 6, 8, and 10.

A second WMC measure assessed children’s sentence span (listening span). The main reason to include a sentence span memory measure was to explore the impact of working memory on the development of math, reading, and spelling. Thus, we adopted the Daneman and Blennerhassett [39] listening span measure and first used it when children were 6 years of age and in their last kindergarten year (t1). The test was repeated at ages 8 and 10 (t2 and t3) and again at ages 18 and 23 (t4 and t5) when the participants were no longer children. Including the listening span test in later assessments enabled us to explore the impact of individual differences in working memory capacity on memory performance in other tasks. In our version of the listening span task, participants first listened to a set of sentences read to them by the experimenter, and then recalled each sentence verbatim. The test was constructed with 75 sentences, three to seven words in length. Each sentence ended in a different word. There were 25 sets of sentences in all, including 5 sets of 1, 2, 3, 4, and 5 sentences. The prerecorded sentences in each set were presented to the participants as a playback from tape. For sets containing more than one sentence, the sentences were read in quick succession. At the end of each set, a tone was emitted that was a cue to recall, verbatim, each sentence in the set. Participants began with the five 1-sentence sets, and were then presented with sets containing increasingly more sentences. Testing terminated when the participants failed all five sentences of a set at a particular level. Subjects were encouraged to repeat the sentences in the order presented, but if they could not recall the order, they were to say back as many sentences as possible in any order.

Participants were given credit for correctly recalling a set as long as they repeated each sentence verbatim, regardless of the order in which they repeated the sentences. In accord with the criterion used by Daneman and Blennerhassett [39], the level at which a participant was correct on at least three out of five sets was taken as a measure of his or her listening span. In addition, the total number of correct sentences and the total number of words reproduced in the correct order were used as dependent variables (for more information on the tests, see [40]). Although our scoring procedure differed from that used in most listening span tasks in that it did not contain WM processing demands, a small pilot study carried out at the beginning of the LOGIC study revealed that the outcome in WM and STM versions of the task was very similar (see Discussion section below). Internal consistency was high for the word span and the sentence span tasks, ranging between 0.78 and 0.85. 

A total score for WMC was calculated for t1 to t5 using the average of the available standard scores. 

#### 2.1.3. Academic Achievement

A task assessing early word and nonword decoding speed was presented in Grade 2 (t2) when children were about eight years of age. The items consisted of four-letter words and pseudowords and were presented on a computer screen. An internal timing device measured children’s responses from the moment of presentation on the screen. A total of 30 words and 30 nonwords were provided. Mean decoding speed was calculated separately for both types of words and for the analyses the average of these scores was used. The decoding speed tasks were given both at the beginning and at the end of second grade. 

Two tests taken from the PISA 2000 (Programme for International Student Assessment) study were presented in the last Wave to assess young adults’ reading comprehension. A text on “Origins of the Moon” was part of the German contribution to reading comprehension in PISA 2000 (cf. [41]). Participants had to read a text about the origins of the moon and to memorize the content as well as possible. After eight minutes reading time, participants were given 13 questions referring to the content of the text, and several shorter items that assessed the domain knowledge as well as their interest in the domain covered by the text, yielding a maximum score of 48.

The second PISA text concerned a story (“Amanda and the Duchess”) that was included in a field trial but not considered for the main PISA 2000 study. This narrative text is about a meeting between Amanda, a young saleslady, and a duchess. Participants read the study, and were then asked to answer five questions about the story, two in multiple-choice format and three using an open format. Correct answers to four of the questions were given one point per item, and answers to the fifth item were given three points, yielding a maximum score of 7 points. Results on both PISA text reading comprehension tasks were combined into a single measure. 

Spelling was assessed more frequently through the course of the LOGIC study. The two spelling tests given in second grade (word dictations, t2) consisted of two partially overlapping versions, one presented at the beginning of second grade and the other shortly before the end of second grade. Each test included about twenty target words that were taken from different sources and were judged to be particularly suited to assess spelling competence in second grade. The spelling tests provided at t3 was more comprehensive (81 words), and was given as sentence dictations. About two thirds of the material consisted of familiar words taken from the official vocabulary list for fourth graders distributed by the Bavarian Ministry of Education. It was assumed that these words were already practiced in the classroom and thus could be mastered by the majority of children. The remaining items were less familiar and consisted of irregular words.

At t4 and t5 (when participants were 18 and 23 years of age), a subtest (“Moselfahrt”) taken from a standardized test of spelling skills (Rechtschreibungstest RT) [42] was used. In this test, a narrative text is read to the participants. Responses are given in a fill-in-the-blank test. An answer sheet provided to participants included parts of the text with empty spaces for a total of 65 target words that participants were to write in. Test norms were available for older children (from age 13 on), adolescents, and adults. For this test and all other spelling measures included in the LOGIC study, the number of correct items was chosen as the dependent variable (see [43], for more information on the reading and spelling tests).

Mathematical competencies were assessed with different tasks at t2, t3, and t4. In Grade 2 (t2), participants had to solve both standard mathematical problems, which were related to school mathematics, and reasoning problems, which were not practiced in school and required use of mathematical strategies. The standard mathematical problems comprised 10 addition and 10 subtraction problems with the results of the addition problems and all subtrahends being smaller than 20. Reasoning problems consisted of another series of addition and subtraction problems. For some of them obviously wrong answers were provided, and for other problems wrong answers were presented that deviated only by one or two from the correct answer. Participants had to decide whether an answer was correct or not as fast as possible. 

In Grade 4 (t3), participants were presented with six mathematical relation problems during each of two assessment sessions. Two circles were cut into 8 or 16 pieces, and a varying number of “slices” were removed. Participants had to decide which of the removed number of slices represented a larger part of a whole circle. These problems were embedded in a story of a merchant selling brown and yellow cheese. In addition, participants were presented with four mathematical word problem such as “How many more dolls does Susan have than Mary?” Here, the task was to choose out of several sentences, containing different information those that need to be put before this word problem in order to being able to solve this problem.

Finally, at t4, when participants were 18 years old, they had to solve a series of mathematical problems that were presented as speed tests and that could be solved by applying techniques of algebra and geometry taught in Grades 8 and 9 in Germany at that time. Altogether, seven numerical problems and 13 word problems had to be solved, and participants were given 5 min for the numerical and 10 min for the word problems. Total scores at t2, t3, and t4 were used as indicators for mathematical competencies of participants.

An overview of all study variables is provided in Table 1 and descriptive statistics are presented in the Appendix A.

### 2.2. Analytic Approach

In a first step, the associations of WMC with the different measures of verbal and fluid intelligence across time will be analyzed and presented. Here, the correlations between WMC and verbal and fluid intelligence for each assessment will be compared with an analytic approach developed by Steiger [44]. Second, regressions analyses will be conducted to predict various measures of academic achievement. Due to the large number of missing data, we decided to use multiple imputations (MI) for these analyses. Consequently, several complete data sets were created by MI, and both correlational and regression analyses were carried out separately for all of them. Finally, the results were averaged allowing for all variances. The MI procedure is conceived of as the gold standard for the treatment of missing data ([45], cf. [46]). 

In all regression analyses, we controlled for participants’ age, sex and socio-economic status (SES) and included intelligence as additional predictor in a subsequent model (Step 1). In Step 2, WMC was introduced in the model, and in models predicting academic achievement at t3 to t5 we also did a second series of regression analyses and included the directly preceding academic achievement as “precursors” before introducing intelligence and WMC. In all models that predicted outcomes at t2, only intelligence and WMC assessed at t1 were included. For the prediction of outcomes at t3, “precursors”, intelligence, and WMC assessed at t2 were included in the models and so on. 

**Figure 1:**
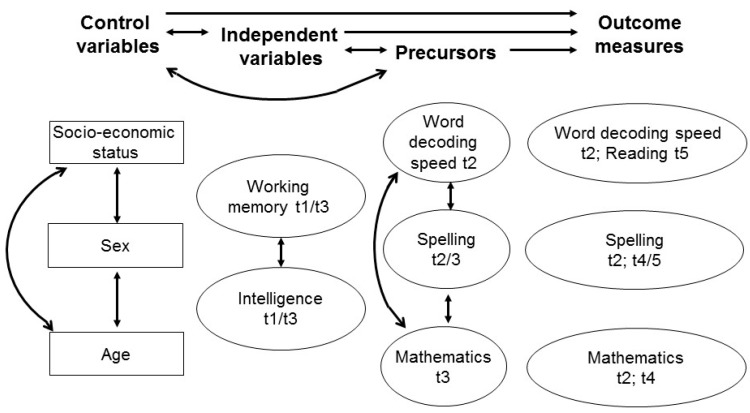
Full-forward model for the prediction of academic achievement by working memory, intelligence and control variables.

In addition to the correlational and regression analyses, we used structural equation modeling to compare outcomes for the different methodological approaches. Here, the “Full Information Maximum Likelihood” (FIML)-method was used (cf. [46]) and the analytic model was specified for all cases, including those cases for which no complete data was obtained. The FIML-method considers all observed values when estimating the model parameters and missing values are ignored for the estimations without reduction of the sample. We followed the suggestions of Marsh, Byrne and Seeshing Yeung [47] and Retelsdorf, Köller and Möller [48] in that we began with “full-forward” SEM models, in which all correlations and all paths from all constructs in each wave to all constructs in subsequent waves are freely estimated. Our complex full-forward models were subsequently pruned and only significant paths remained in the model. To reduce alpha inflation due to multiple testing, we adjusted the alpha level based on the number of significance tests and the mean latent correlations between factors (see [49] for the adjusted Bonferroni-correction procedure). Figure 1 shows the theoretical basis for our SEM models with correlations between and within all control, independent and precursor variables and prediction paths from all these variables to all outcome measures.

Three models were tested: (1) Prediction of academic achievement at t2 by participants’ age, sex, and SES as well as intelligence and WMC assessed at t1; (2) Prediction of academic achievement at t4/t5 by participants’ age, sex, and SES as well as intelligence and WMC assessed at t3; and (3) Model 2 with the inclusion of academic “precursors” assessed at t2/t3. Due to the adjusted Bonferroni-correction procedure, we tested against α = 0.02 in model 1, α = 0.0188 in Model 2, and α = 0.0129 in Model 3. The “Incremental Fit Index” (IFI), the “Tucker-Lewis-Index” (TLI), the “Comparative Fit Index” (CFI), the “Root Mean Square Error of Approximation” (RMSEA), and the ratio of Chi2 and degrees of freedom (df) will be reported. To create latent variables for spelling, two subsequent assessments of spelling were combined. This approach was possible due to the very high correlations (r_t2/t3_ = 0.74; r_t4/t5_ = 0.82) indicating the stability of spelling abilities. In a final step, cascading models were tested in which intelligence acted as mediator between working memory and outcomes.

## 3. Results

Table 2 shows the results for the correlational analyses of WMC and non-verbal and verbal intelligence across t1 to t5.

All correlations between WMC and intelligence were positive and mostly significant. Non-significant correlations were found only for WMC assessed at t4 and t5 due to the larger measurement errors in the MI approach (significant correlations only were found in the original data). When comparing the cross-sectional correlations with non-verbal vs. verbal intelligence, indeed slightly greater correlations were found for the association of WMC with verbal intelligence. However, these differences were not large and they were not significant with the exception of t2 (*p* < 0.05).

Results of all regression analyses are presented in Table 3. Here, the total variance R² explained for the criterion variable in the final model as well as the variance additionally explained after introducing either preceding academic achievement (“precursors” Step 1), intelligence (Step 2) or WMC (Step 3) will be presented as well as the t-value for the predictors.

Both WMC and intelligence explained a significant amount of additional variance of academic achievement at t2 after participants’ age, sex, and SES had been controlled for. However, when previous academic achievement was included in the models to predict later academic achievement, intelligence was only a significant predictor for reading comprehension at t5 (explaining 14% of additional variance) and for mathematics at t3 (explaining 19% of additional variance), and WMC was not a significant predictor at all. Academic precursors were most important for later spelling abilities and for mathematics at t4 when participants were about 18 years of age, explaining between 11% and 45% of the variance in the criterion variables.

**Figure 2:**
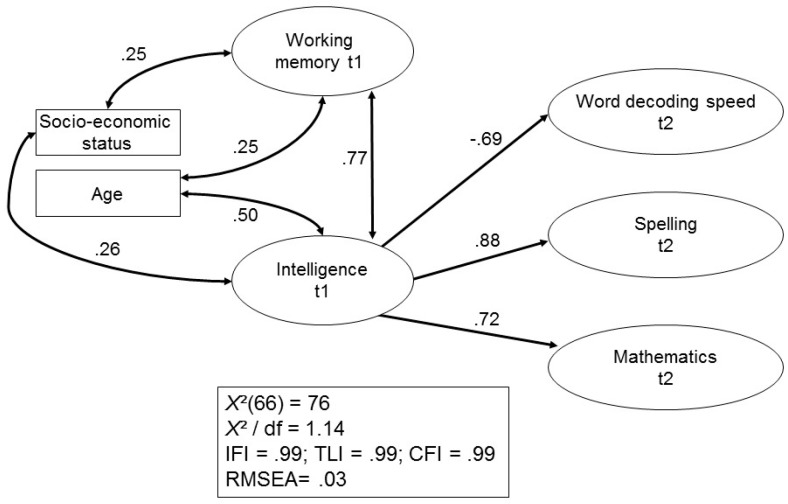
Prediction of academic achievement at t2 by working memory, intelligence and control variables.

In a final step, SEM was also used to predict academic outcomes. Figure 2 shows the result for the prediction of academic achievement at t2.

**Figure 3:**
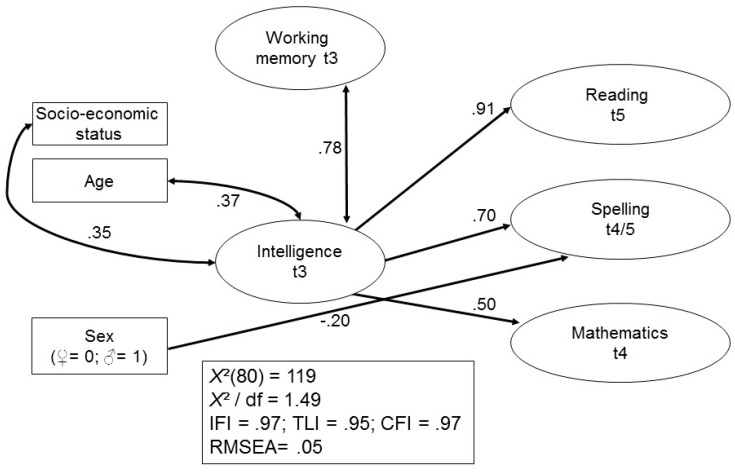
Prediction of academic achievement at t4/t5 by working memory, intelligence and control variables.

Older participants and participants with a greater SES showed better results in the WMC and intelligence assessments. However, no control variable was a significant predictor of academic achievement at t2. WMC and intelligence were highly intercorrelated as latent variables, and intelligence was the only significant predictor of all academic outcomes in Grade 2. The model fit characteristics indicate a very close and very good fit to the raw data. Figure 3 shows the same model for the prediction of academic achievement at a later stage in development (i.e., at t4/t5). Again, a very close and good fit of the model to the raw data was found.

**Figure 4:**
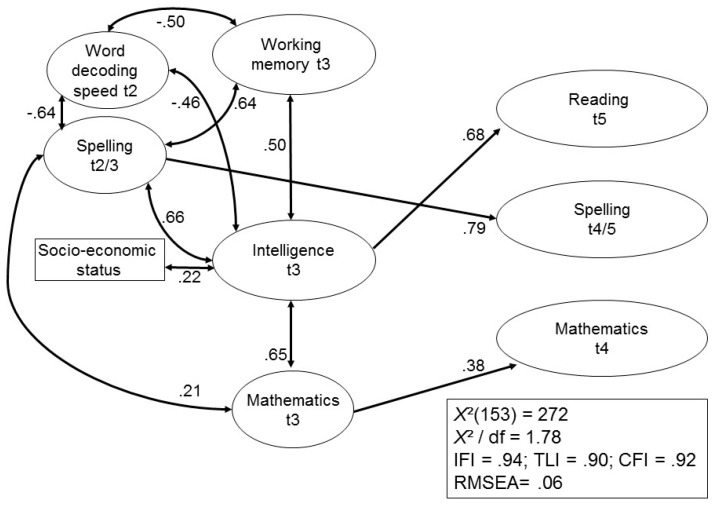
Prediction of academic achievement at t4/t5 by working memory, intelligence, precursors and control variables.

In early adulthood, females were significantly better than males in spelling. Again, only intelligence was a significant predictor of academic outcomes, even though WMC and intelligence were highly associated latent variables at t3. Figure 4 shows the same model, when academic achievement at t2/t3 were introduced.

A good fit of the model to the raw data was found. With the exception of mathematics and WMC at t3 and mathematics at t3 and word decoding speed at t2, WMC, intelligence and all measures of early academic achievement were significantly correlated, with correlations yielding scores mostly of about 0.45 and above. In regard to the control variables, only the correlation between SES and intelligence remained significant, and child age and sex were omitted from the model. Spelling in early adulthood was predicted by earlier spelling abilities only and mathematical competencies at t4 were predicted by mathematics at t3 only. Intelligence remained a significant predictor for later reading competencies, whereas WMC did not predict any of the outcome measures. 

In a final step of analysis, we tested the cascading SEM model suggested by Demetriou and colleagues [20], in which IQ acts as a mediator between children’s WM and academic achievement. Consequently, correlations between WM and IQ were replaced by paths predicting IQ via WM in all models. Fit indices for all three alternative Models 1a, 2a, and 3a were slightly worse, but comparable to the original Models 1, 2 and 3, and WM acted as a strong predictor of IQ. Verbal IQ predicted the same measures of academic achievement as in the original models, and also significantly predicted mathematical competencies at t4 in Model 3a. In accord with former research, IQ thus mediated the impact of working memory on children’s academic outcomes (cf. [20,31]). 

## 4. Discussion

One of the major goals of the present secondary analysis was to explore whether the impact of IQ and WMC on academic achievement would be different at different stages of child and adolescent development. One of the advantages of the LOGIC study is that the sample was followed from early childhood to emerging adulthood, and that measures of IQ (both verbal and non-verbal) and WMC were taken throughout the course of the study. We knew from previous data analyses on the LOGIC sample that both IQ components as well as the core WMC measure (listening span) increased significantly over time until adolescence [40,37]. In all cases, a nonlinear growth model fit the data best, suggesting a negatively accelerated growth curve and indicating that the growth curve flattened at later measurement points. In fact, verbal IQ and WMC did not improve between the ages of 18 and 23 years of age, whereas nonverbal IQ still increased during this period. We also know from previous data analyses that stability over time was substantial and comparable for the IQ and WM variables [3,40]. The new aspect of the present re-analysis concerned the issue whether the predictive power of the WM and IQ variables regarding academic achievement would be different at different occasions. Thus far, only the study by Alloway et al. [26] had tackled the issue using a longitudinal design. 

What were the major findings of the present secondary analysis? First, our analyses confirmed that the indicators of verbal and nonverbal IQ were reliably and positively correlated with the WM measures on most occasions. However, as can be seen in Table 1, the synchronous correlations among the observed variables were not as large as reported in most cross-sectional studies, varying between r = 0.17 and 0.40. Relationships were considerably higher for the latent IQ and WM variables, which is in accord with previous findings (e.g., see [28]). 

Did we find different prediction patterns at different developmental stages? According to the regression analyses, IQ and WMC explained a significant amount of variance in all measures of academic achievement at the very beginning of school even after participants’ age, sex and SES were controlled for. Both IQ and WMC were significant predictors of early spelling, whereas word decoding speed at t2 was significantly predicted by WMC only in regression analysis. In regard to early mathematics, neither IQ nor WMC were significant predictors when both variables were introduced into the same model, and they explained only about 5% of the variance in this outcome variable. 

One possible reason for this unexpected finding is that the mathematics tasks assessed at the beginning of elementary school measured more general mathematical reasoning than direct outcomes of school instruction [50]. The focus changed to some extent during later assessments when arithmetic word problems, mathematical relation problems, and numerical problems were presented. Not surprisingly, considerably more variance in mathematics performance (20%) could be explained by IQ and WMC when children were tested at age 10 (t3), with IQ turning out to be the most powerful predictor, explaining 23% of the variance in the dependent measure, even when the control variables and previous mathematical competencies were controlled for. 

Similarly, IQ was the best and the only significant predictor of reading comprehension in early adulthood. This finding was unexpected, given that we hypothesized that preceding educational achievement should have a greater impact on later academic achievement than IQ and WMC (cf. [27]). However, there is an obvious explanation for this result. Whereas IQ and WMC at t4 (and thus assessed at the age of 18) were introduced into the regression analysis predicting reading comprehension at t5, no “precursors” of reading were assessed at t3 or t4. Consequently, word decoding speed assessed at t2 (age 8 years) was included into the model as “precursor” instead, a measure assessed ten years earlier than IQ and WMC. It should be noted that this finding is in accord with the model of WMC developed by Demetriou et al. [20] and thus received support in a previous study.

When turning to the final assessments of spelling (t5) and mathematics (t4), the expected pattern was found: After the immediate precursors were included in the models, neither IQ nor WMC explained significantly additional variance in the outcome measures. It should be noted again that the math tasks used at t4 were not similar to those actually included in the typical math curriculum, which may explain the fact that the proportion of variance explained in the criterion variable was not particularly impressive.

We also conducted exploratory regression analyses to predict the last assessments of spelling and mathematics and omitted the precursors in both models. Here, the impact of IQ was significant and stronger than that of WMC in both cases, whereas WMC was a non-significant predictor. This finding is contrary to some previous findings (e.g., [26,22]), however, it aligns with other studies (e.g., [25]). 

Did the pattern of findings change much when latent variable structural equation modeling (SEM) was used in a next step? Of course, results for the regression and the SEM models were not directly comparable given that the three outcome measures were separately analyzed in the regression models but simultaneously considered in the SEM models. Nonetheless, findings for the early phase and the last assessment period are comparably similar, as shown in Figure 2 and Figure 3. IQ and WMC were very closely related at both time points, and IQ had a significant and strong impact on reading abilities, spelling, and math, regardless of measurement point. These findings resemble those reported by Lee et al. [28] who found that only IQ directly predicted their outcome measure (i.e., algebraic proficiency) in SEM models compared to somewhat different results in the regression analyses. They are also in accord with those by Giofre et al. [31] indicating that intelligence can predict reading and math achievement above and beyond the effect of WMC. Our findings differed from those reported by Alloway et al. [26] in that IQ played a more important role for academic achievement than WMC.

An aspect not considered in the various studies described above concerned the role of domain knowledge, that is, the impact of preceding performance regarding reading, spelling, and math on subsequent assessment of these subject matters. The findings depicted in Figure 3 indicate that spelling and math assessed at the ages of 8/10 significantly predicted spelling and math at the ages of 18/23, thus confirming the important role of preceding domain knowledge on subsequent development in the respective domains [43]. Our results thus indicate that domain knowledge increasingly affects academic achievement over time and may be more important than more general abilities such as IQ or WMC. 

Whereas word decoding speed had no impact on reading comprehension assessed at the age of 23, IQ assessed at age 10 directly predicted reading at the later occasion. Overall, our findings indicate that both IQ and WM influence educational achievement in early and later stages of development and that IQ seems to play an even more important role in this context. The role of IQ seems particularly impressive, supporting previous findings by Deary et al. [1] and Sternberg, Grigorenko, and Bundy [51]. They are not in accord with findings by Alloway et al. [26] and Gathercole et al. [22] which indicated that WM is a more powerful predictor of academic performance than general IQ.

Our tests of the cascading model suggested by Demetriou and colleagues [20] as well as by Giofre et al. [31] revealed that the data were also in accord with the assumption that IQ acts as a mediator between children’s WM and their academic achievement. IQ was also a significant predictor of mathematical competencies at t4 in Model 3a. Overall, fit indices for the cascade model were comparable to those of the original model, but not significantly better. Further studies are needed to clarify this issue.

## 5. Limitations

One of the problems related this kind of research concerns the balance between selected indicators of IQ and WM. For instance, whereas Alloway et al. [26] considered several indicators of WMC, they only included a few selected subtests of IQ in their study. This procedure may at least partially account for the pattern of findings and the fact that WM was a more powerful predictor than IQ. One may also argue in our case that only a few measures of STM were considered, whereas several IQ tests were included in the LOGIC study. Another problem issue concerns the fact that our memory measures seem to tap STM and not so much WM given that the assessment of the sentence (listening) span deviated from the traditional assessment procedure. That is, whereas the original listening span requires that individuals perform a specific action, selecting the last word of each sentence while maintaining all information in the phonological store, the participants in the LOGIC study were asked to recall as many sentences as possible without performing additional operations on the stimulus material. Accordingly, this modified task seems to tap STM more than WM in the tradition of the Baddeley and Hitch [8] model.

Another problem that cannot be avoided in longitudinal studies covering the age range from early childhood to young adulthood is that different measures had to be used at different time points. Thus, it was not possible to use vertical scaling to investigate growth and change in the various measures over time. 

An additional problem concerns the fact that we used an absolute credit score in the sentence span task, counting trials as correct only when all words of a sentence were memorized. A problem with the absolute scoring method is that the difficulty of a span item may vary on many dimensions, thus threatening span reliability across different tasks. As noted by Giofre and Mammarella [52], also considering the trials in which participants provided only a partial response yields a more robust of children’s actual memory span. 

Although there is no doubt that the traditional version of the sentence span tasks and the version used in our study differ from a qualitative point of view, the difference between the two versions may not be as large as one would expect. When piloting the memory tasks of the LOGIC study more than 30 years ago, a small cross-sectional study was carried out where participants of different ages received both versions of the sentence span task in a counterbalanced order. Given that the correlations between the two versions were substantial, exceeding r = 0.8 in most age groups, our impression was that the rather complex STM version of the task lead to comparable results. Our interpretation of this finding was that the executive (WM) component in the traditional version of the task is not particularly strong: participants who are able to maintain many words in their memory store for a couple of seconds are well able to select the last word of a given sentence, and vice versa. Given that we were interested in increasing the variance of outcomes in this task, particularly with preschool children at the very beginning of the study, we decided to work with the STM version.

Related to this, it seems possible that our sentence span task addresses the “episodic buffer” in Baddeley’s [53] WM model, which is supposed to be a limited capacity system that depends heavily on executive processing but that differs from the central executive in that it is primarily concerned with the storage of information rather than with attentional control. According to Alloway, Gathercole, Willis and Adams [54] the recall of spoken sentences should be a suitable paradigm to investigate the episodic buffer because repeating sentences involves the integration of information from temporary memory (to support the verbatim recall of individual words and their order) with the products of semantic and syntactic analysis by the language processing system. Thus, the sentence span measure was viewed as a way of capturing how a child may integrate information from the phonological loop with knowledge and analyses carried out on the sentences by the language processing system. 

## 6. Conclusions

The present study provided initial support for the assumption that both IQ and WM are important predictors of academic achievement, as indicated by the result of various regression analyses. However, according to the outcome of more comprehensive SEM analyses, the impact of IQ on achievement seems more direct and stronger than that of WM, regardless whether early or late periods of schooling are considered. There was no evidence supporting the assumption that the predictive power of IQ and WM was different for different aspects of academic learning (reading versus math). Most notably, however, we found that individual differences in IQ and WM no longer played a significant role in the prediction of subsequent academic performance when the impact of previous domain knowledge was additionally estimated in the SEM models. One implication of this finding is that IQ and WM may be important predictors of achievement during early stages of schooling when domain knowledge is not that well developed, but that they lose their impact at later schooling periods. To clarify this issue, future research on the topic should be based on longitudinal designs that not only provide several representative measures of IQ and WM during early and later assessment periods but also include relevant indicators of domain knowledge at each time point in order to estimate the size of specific IQ and WM effects and their changes over time. 

## Figures and Tables

**Table 1:** Overview of the assessments and corresponding ages of the test persons, the number of participants tested at least with one of the tests, and the tests used during assessments (English abbreviations in parentheses).

**Time**	1	2	3	4	5
**Average age in years**	6	8	10	18	23
**Number of participants tested**	208	197	193	174	151
**Verbal IQ measures**	HAWIVA (WPPSI)	HAWIK-R (WISC)	HAWIK-R (WISC)	HAWIE-R (WAIS)	HAWIE-R (WAIS)
**Nonverbal IQ measures**	CMMS	CMMS	CFT	CFT	CFT
**Sentence span**	Daneman	Daneman	Daneman	Daneman	Daneman
**Word span**	Case	Case	Case	-	-
**Reading**	-	Word decoding speed	-	-	Text comprehension Fast reading
**Spelling**	-	Dictation	Dictation	Dictation	Dictation
**Mathematics**	-	Arithmetical problems	Arithmetical and relation problems	Arithmetical problems	-

Note: WPPSI = Wechsler Preschool and Primary Scale of Intelligence; WISC = Wechsler Intelligence Scale for Children; WAIS = Wechsler Adult Intelligence Scale; CMMS = Columbia Mental Maturity Scale; CFT = Culture Fair Intelligence Test; Daneman = Daneman’s Sentence Span Task; Case = Memory span for words (unconstrained and serial word span).

**Table 2:** Correlations between WMC and non-verbal and verbal intelligence for t1 to t5 (cross-sectional correlations in bold).

	Non-Verbal Intelligence t1	Verbal Intelligence t1	Non-Verbal intelligence t2	Verbal Intelligence t2	Non-Verbal Intelligence t3	Verbal Intelligence t3	Non-Verbal Intelligence t4	Verbal Intelligence t4	Non-Verbal Intelligence t5	Verbal Intelligence t5
Working memory t1	0.328 ***	0.342 ***	0.301 ***	0.411 ***	0.371 ***	0.428 ***	0.319 ***	0.284 **	0.287 **	0.265 **
Working memory t2	0.223 *	0.269 **	0.255 **	0.404 ***	0.357 ***	0.423 ***	0.254 **	0.299 **	0.282 ***	0.256 **
Working memory t3	0.246 *	0.236 **	0.227 *	0.387 ***	0.325 ***	0.401 ***	0.269 **	0.231 **	0.192 *	0.236 *
Working memory t4	0.209 *	0.158 *	0.204	0.221	0.237 **	0.150	**0.198**	0.296 ***	0.165 *	0.264 *
Working memory t5	0.164	0.190	0.220	0.250	0.225	0.207	0.210	0.325	**0.170**	**0.248**

* *p* < 0.05; ** *p* < 0.01; *** *p* < 0.001.

**Table 3:** Results of regression analyses to predict achievement at t2, t3 or t4/5 by previous academic achievement (Step 1), intelligence (Step 2), and working memory (Step 3) at t1, t2 or t3/4 controlled for participants’ age, sex, and SES.

	Spelling t2	Spelling t3	Spelling t5	Word Decoding Speed t2	t5 Reading Comprehension	Mathematics t2	Mathematics t3	Mathematics t4
Total R² (final model)	0.30 ***	0.58 ***	0.48 ***	0.16 ***	0.29 ***	0.08 **	33 ***	16 ***
ΔR^2^ (Precursors $)	-	0.45 ***	0.36 ***	-	0.09 ***	-	0.05**	0.11 ***
ΔR^2^ (Intelligence)	0.10 ***	0.02 *	0.01	0.05 **	0.14 ***	0.03 *	0.19 ***	0.00
ΔR^2^ (Working memory)	0.04 **	0.01	0.03	0.05**	0.02*	0.02*	0.01	0.00
	t	T	t	t	t	t	t	t
Precursors $	-	7.10 ***	4.05 **	-	−3.542 **	-	3.21 **	4.72 ***
Precursors $	-	5.12 **	2.65 *	-	−2.03	-	1.94	3.58 ***
Intelligence #	4.31 ***	1.70	0.45	−2.47 *	4.38 ***	2.34 *	7.16 ***	0.71
Precursors $	-	4.72 **	2.78 *	-	−1.88	-	1.75	3.35 **
Intelligence #	3.05 **	1.32	0.30	−1.33	4.00 **	1.38	6.00 ***	0.53
Working memory #	2.95 **	1.88	0.57	−3.10 **	1.48	1.91	1.62	0.69

* *p* < 0.05; ** *p* < 0.01; *** *p* < 0.001. # The preceding assessment of Intelligence and Working memory was used as predictor (i.e., t1 for the prediction of t2, t2 for t3, t3 for t4, and t4 for t5). $ Precursors for Spelling t3 and t5: Spelling t2 and t4; precursors for Reading Comprehension and Fast reading: Word decoding speed t2; precursors for Mathematics t3 and t4: Mathematics t2 and t3. ΔR^2^ is the additionally explained variance after the variable in the parenthesis was added to the model.

## Appendix A.

**Table A1:** Descriptive Statistics for All Study Variables (Minimum, Maximum, Mean, Standard Deviation, Skewness, Kurtosis, and Reliability).

Measures	Time	N	Min	Max	M	SD	Skewness	Kurtosis	Reliability#
Nonverbal intelligence	t1	189	3	82	58.98	8.80	−1.35	7.89	
	t2	194	44	85	68.57	7.18	−0.48	0.91	
	t3	193	11	42	29.44	5.65	−0.34	−0.01	0.69
	t4	174	79	147	120.49	14.48	−0.15	−0.68	0.70
	t5	145	18	46	39.68	4.73	−1.36	2.50	0.74
Verbal intelligence	t1	207	21	64	46.36	8.41	−0.42	0.20	0.74
	t2	196	11	73	35.97	13.39	0.33	−0.60	0.78
	t3	192	19	102	62.09	13.94	−0.08	0.17	0.77
	t4	174	9	29	19.25	4.08	−0.10	−0.76	0.76
	t5	151	8	31	21.70	4.51	−0.44	−0.03	0.79
Serial word span	t1	207	0	6	3.47	0.97	−1.12	3.66	
	t2	197	0	6	4.15	0.88	−1.03	3.52	
	t3	187	2	7	4.78	0.86	−0.02	0.55	
Unconstrained word span	t1	207	1	6	3.84	0.80	−0.21	0.08	
	t2	197	3	6	4.46	0.82	−0.13	−0.52	
	t3	187	2	7	5.28	0.90	−0.37	0.15	
Total nr. of correct words (listening span)	t1	207	20	211	99.39	36.99	0.47	0.13	
	t2	195	40	238	141.86	45.66	0.10	−1.04	
	t3	193	61	232	157.32	43.09	−0.09	−1.18	
	t4	170	68	339	226.05	53.80	0.23	−0.19	
	t5	151	65	340	214.35	55.57	0.10	0.13	
Total nr. of correct sentences (listening span)	t1	208	2	38	14.05	6.63	0.69	0.69	0.83
	t2	195	5	56	21.56	8.58	0.48	0.15	0.85
	t3	193	6	40	24.83	8.14	0.02	−0.99	0.84
	t4	170	9	61	38.09	9.72	0.20	0.06	0.98
	t5	151	7	59	36.54	10.21	0.07	0.22	0.98
Word decoding speed	t2	186	2	9	4.29	1.51	1.22	1.26	0.83
Text comprehension (Moon)	t5	150	11	48	34.53	8.07	−0.71	−0.01	0.69
Text comprehension (Duchess)	t5	150	0	7	4.70	1.76	−0.34	−0.84	0.66
Spelling	t2	194	2	18	11.20	4.01	−0.46	−0.59	0.84
	t3	192	17	60	49.81	8.22	−1.29	1.67	0.89
	t4	174	15	62	46.04	8.10	−0.53	0.53	0.86
	t5	151	15	63	46.98	7.92	−1.09	2.00	0.88
Standard mathematical problems	t2	157	10	26	20.75	3.54	−0.81	0.23	
Reasoning mathematical problems	t2	157	5	18	12.80	2.83	−0.54	−0.18	
Mathematical relation task 1	t3	193	0	6	3.24	2.16	0.04	−1.62	0.82
Mathematical relation task 2	t3	193	0	6	3.56	2.13	−0.21	−1.61	0.82
Word problems	t3	193	0	4	2.03	1.01	−0.36	−0.62	
Numerical problems	t4	173	0	6	1.55	1.18	0.70	0.43	0.54
Word problems	t4	174	0	9	3.25	1.80	0.81	0.57	0.64

# Note: Reliabilities (Cronbach’s alpha) in this table are based on data in the LOGIC study (e.g., subscale or item level) and thus differ from those reported in the text. For nonverbal intelligence at t1 and t2, mathematical problems at t2 and word problems at t3, and for working memory, only sum scores were available in the data of the LOGIC study. For the total scores of working memory t1 to t5, reliabilities (Cronbach’s alpha) are provided in the rows of the listening span for sentences based on the combination of all sum scores of working memory tasks (listening span (words and sentences) and serial and unconstrained word span).
